# Shear Performance of Epoxy Joints in a Precast Bridge Deck Considering Constraint Effects

**DOI:** 10.3390/polym15153327

**Published:** 2023-08-07

**Authors:** Jiangtao Zhang, Hongjie Wang, Yanjiang Yu, Kaidi Zheng, Zhixiang Zhou, Jinlong Jiang

**Affiliations:** 1 School of Civil Engineering, Chongqing Jiaotong University, Chongqing 400074, China; zhangjiangtao@cqjtu.edu.cn (J.Z.); wanghongjie@mails.cqjtu.edu.cn (H.W.); 2 China Merchants Chongqing Testing Center for Highway Engineering Co., Ltd., Chongqing 400060, China; 3 Chongqing Jiaduo Highway Design Consulting Co., Ltd., Chongqing 400039, China; 4 Department of Civil Engineering, School of Civil and Transportation Engineering, Shenzhen University, Shenzhen 518060, China; zhixiangzhou@szu.edu.cn

**Keywords:** assembled steel composite bridges, epoxy joints, single shear test, constraint effect, shear performance

## Abstract

The joint form plays a vital role in the rapid assembly of precast bridge decks for steel–concrete composite bridges. Existing research primarily focuses on studying the shear performance of joints through direct shear tests, which is insufficient to fully reflect the mechanical behavior of joints under the constraint of prefabricated bridge deck panels during actual vehicular traffic. Considering situations such as vehicle loads and external forces acting on precast bridge decks, this study investigates the shear performance of epoxy joints under constraint through an improved shear test. The influence of constraint force, shear key details and interface defects on the shear performance of epoxy joints is investigated. The results reveal that the shear test method employed in this study can realistically reflect the shear performance of epoxy joints in precast bridge decks. Both active and passive constrained epoxy joint specimens exhibited no interface cracks, and their failure modes were identified as shear failure between mid-span supports. Compared with passive constraint, the shear-bearing capacity of epoxy joint specimens under active constraint was increased by 86.1~130.6%. Among the epoxy joint specimens with depth–height ratios of 15/110, 25/110, 35/110 and 45/110, the joint with a depth of 35 mm demonstrated the highest shear strength. Furthermore, the shear performance of epoxy joints significantly deteriorated when the interface defects exceeded 30%, resulting in the failure mode transforming from shear failure to interface failure.

## 1. Introduction

Assembled steel–concrete composite bridges are well suited for the construction demands of industrialized and standardized production, offering advantages such as high construction safety, easy construction control and minimal environmental disruption, making them a promising bridge structural system [1,2,3,4,5]. Joints, as crucial elements for connecting precast bridge decks, greatly influence on-site assembly efficiency, bridge load-bearing performance and deck durability [6,7,8]. However, traditional wet joints face three main limitations. Firstly, the dense and complex arrangement of reinforcement increases fabrication difficulties, significantly reducing assembly efficiency [9,10]. Secondly, the “point contact” achieved by coarse aggregate enrichment between cast-in-place concrete and precast concrete creates a low-strength “weak layer”, deteriorating the mechanical performance of joints [11,12,13]. Lastly, uneven shrinkage due to the age difference between cast-in-place concrete and precast concrete leads to crack formation and reduces the durability of the bridge deck [14]. Considering the low assembly efficiency, inadequate interface bonding and poor durability performance of traditional wet joints, it is necessary to propose a joint form that is simple and efficient in construction and ensures sufficient interface bonding.

The joints of precast bridge panels require a simple, reliable, high-efficiency assembly within a shorter construction period considering the significant demand for assembling modular precast bridge panels. Epoxy resin, a versatile polymer substance, holds potential as an ideal joint material because of its exceptional strength and rigidity and superior adhesion, coupled with notable adjustability and processability. Given these characteristics, the employment of epoxy resin as a joint filler in bridge decks is a suitable and effective solution. The application of epoxy adhesive within the joint can fill small defects and eliminate areas of high contact stress [12,15]. However, the discontinuities at the joints have a significant impact on the structural performance of precast bridge decks, resulting in the absence of shear strength provided by concrete aggregates and reinforcement at the interface between new and old concrete. Shear transfer heavily relies on the material and bonding properties of epoxy [16]. Therefore, as vital load-bearing constituents in assembled steel–concrete composite beam bridges, epoxy joints have been the key research focus for many scholars. Their comprehensive investigations into the mechanical performance of epoxy joints aim to indicate their suitability and effectiveness.

The shear performance of dry joints with shear keys and epoxy joints was compared by Gopal et al. [17]. The results indicated that the application of epoxy adhesive to dry joints could enhance shear-carrying capacities. Zhang et al. [18] investigated the shear performance of full-scale multiple-key epoxy joints under direct shear. It was found that an increase in epoxy adhesive thickness resulted in smoother shear stress transfer, as well as more uniform stress distribution on the shear surface. Yuan et al. [19] studied the shear performance of epoxy joints with shear keys and observed that different key shapes, angles, internal reinforcement layouts and reinforcement methods influenced the shear strength, failure mode and ductility of the joints. Chen et al. [20] conducted experimental research and numerical analysis, revealing that epoxy bonding can improve the shear capacity of single-key joints in ultra-high-performance concrete. However, increasing the number of shear keys had a relatively limited effect on the enhancement of joint-bearing capacity under higher confining stress. Meng et al. [21] conducted push-off tests on nine epoxy joints and two dry joints. The results showed that epoxy joints exhibited less ductility compared with dry joints but had a significant advantage in terms of stiffness. Pan et al. [22] performed direct shear tests on epoxy joints with different numbers of shear keys and observed that an increase in the number of shear keys resulted in increased shear stiffness and shear strength in the joints. Zou. et al. [23] compared the mechanical properties of dry joints and epoxy joints under direct shear loading. The results indicated that the shear-carrying capacity of dry joints linearly increased with an increasing number of shear keys, whereas it had a negligible impact on the shear capacity of epoxy joints. Furthermore, the epoxy joint cracking load was the ultimate load and exhibited obvious brittle damage.

Existing studies have focused primarily on immediate shear performance, overlooking long-term behavior and durability. Standardized guidelines and codes for epoxy joints are lacking, hampering comparability and reliability. Practical challenges such as construction ease, time and quality control during installation require consideration. Failure modes, repairability and maintenance requirements remain inadequately explored. Alternative joint designs and materials demand further investigation to enhance overall performance and cost-effectiveness. Addressing these gaps will advance the implementation and understanding of epoxy joints in precast bridge panel assemblies. To adequately consider the effects of vehicle loads and external forces on the precast bridge deck panels, it is necessary to employ appropriate experimental methods to investigate the shear performance of epoxy joints under constraint conditions. Therefore, this study proposes a high-strength, corrosion-resistant and well-adhesive epoxy joint and investigates the shear performance of joints under constraint conditions through an improved shear test. Finally, the effect of different constraint forces, shear key details and interface defects on the shear performance of the specimens is discussed based on a finite element analysis.

## 2. Test

### 2.1. Specimen Design

**Figure 1:**
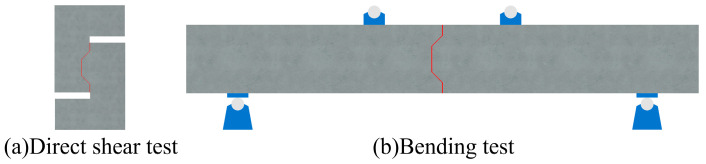
Different loading methods: (**a**) schematic of the direct shear test; (**b**) schematic of the bending test.

As a critical constituent of prefabricated steel–concrete composite beam bridge decks, joints exhibit complex mechanical behaviors that necessitate a suitable testing method to evaluate their mechanical performance. Currently, mechanical performance testing on joints is generally classified into two main categories: direct shear testing [22,24] and bending testing [25,26], as illustrated in Figure 1. Direct shear testing exclusively focuses on the shear properties of the joint, which disregards the restraint effect of the precast concrete plates surrounding the joint. On the other hand, bending tests treat the joint as an integral component of the overall structure, making it difficult to study the mechanical behavior of the joint in detail.

**Figure 2:**
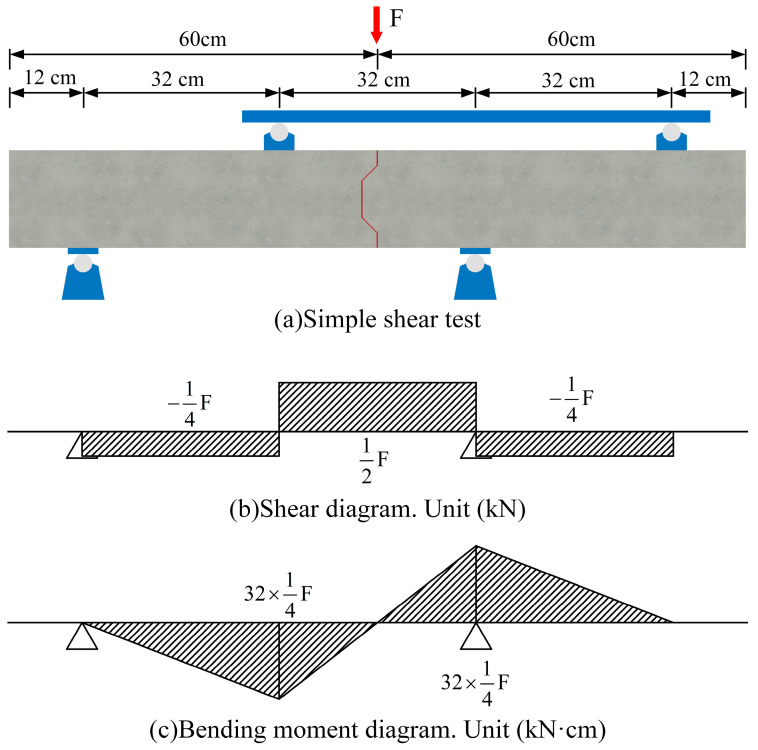
Details of single-shear test: (**a**) schematic of the single-shear test; (**b**) shear diagram; (**c**) bending moment diagram.

Thus, a single-shear loading method is proposed in this study that considers the restraint effects of the precast concrete plates and regards the joint as the primary structural component, as illustrated in Figure 2. The proposed testing method can assess the joint’s shear performance and simultaneously analyze the damage progression of the precast concrete plates surrounding the joint.

**Figure 3:**
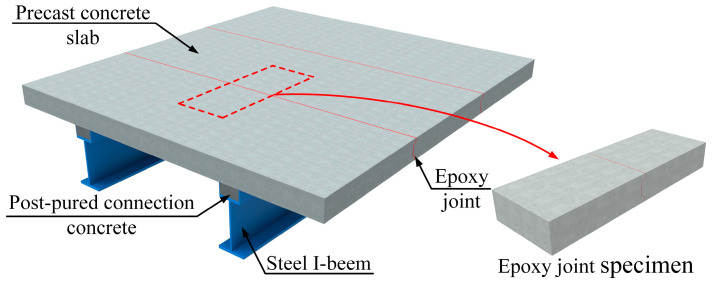
Schematic of the test specimen used in this study.

**Figure 4:**
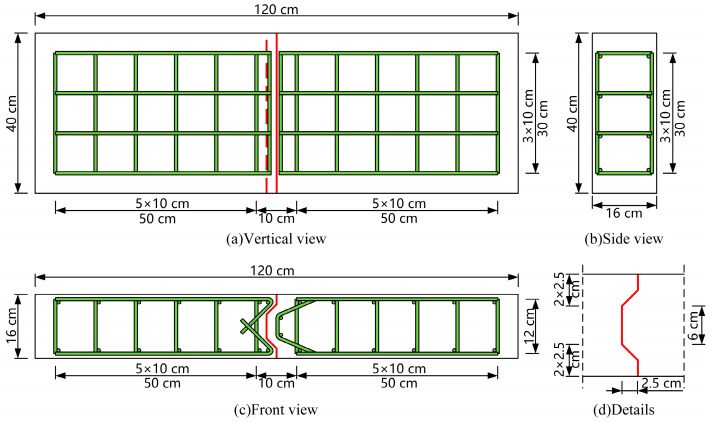
Detailed dimensions of specimens.

This study aimed to investigate the shear performance of epoxy joints. Two epoxy joint specimens were designed, and the relationship between the specimens and a bridge deck system is illustrated in Figure 3. The dimensions of the specimen construction were 120 cm × 40 cm × 16 cm, and the diameter of the hoop reinforcement in the reinforcement configuration was 6 mm, while the diameters of other reinforcements were 8 mm. The detailed dimensions of the specimen are indicated in Figure 4.

Table 1 presents the design parameters of the two specimens in detail. The letter “J” represents the joint designed in this study, “E” denotes the epoxy, and “0” and “2”, respectively, indicate that the stress constraints at the two ends of the prefabricated concrete slab were 0 MPa and 2 MPa.

### 2.2. Material Properties

#### 2.2.1. Epoxy

The epoxy used in the experiment was CFSR-A/B produced by the Carben Chemicals Group (Carbon Technology Group Co Inc., Tianjin, China). The adhesive was mixed in a 2:1 mass ratio of CFSR-A and CFSR-B and stirred evenly until no color difference was observed before use. The mechanical properties of the epoxy were tested in accordance with the test methods for the properties of the resin casting body (GB/T 2567-2021 [27]) in Table 2. $f_{te}$ denotes the tensile strength; $f_{ce}$ is the compressive strength; and $E_{te}$ is the tensile elastic modulus.

#### 2.2.2. Normal Concrete and Reinforcement

The prefabricated concrete slabs were made of C50 concrete, and the strength grade of the reinforcement was HRB400. The material properties of the concrete and reinforcements were evaluated according to the standard test method for the performance of ordinary fresh concrete (GB/T 50080-2002 [28]) and Metallic Materials—Tensile Testing—Part 1: Method of Test at Room Temperature (GB/T 228.1–2010 [29]), respectively. The measured mechanical properties are summarized in Table 3.

### 2.3. Specimen Preparation

**Figure 5:**
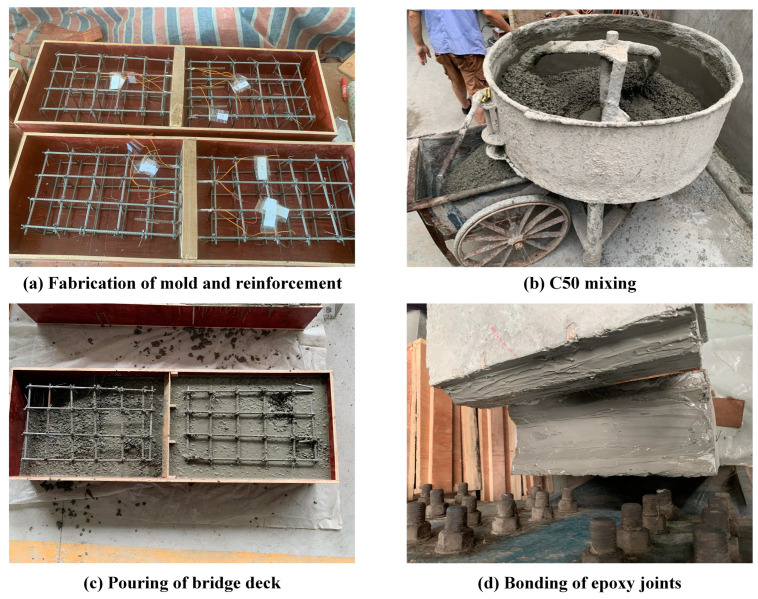
Construction process of the specimens: (**a**) fabrication of mold and reinforcement; (**b**) C50 mixing; (**c**) pouring of bridge deck; (**d**) bonding of epoxy joints.

Figure 5 depicts the fabrication process of the precast concrete slab and joints for both specimens. In Figure 5a, the formwork and reinforcement were both constructed, following which, the reinforcement was set in the formwork to prepare for casting. C50 concrete was mixed and poured into the formwork, as demonstrated in Figure 5b,c, and then cured for seven days until the precast concrete slab achieved sufficient strength. Figure 5d shows that epoxy was applied to the joints, and adequate pressure was exerted at both ends of the precast concrete slab to ensure effective bonding. Following a natural curing period of seven days, the specimens were produced and ready for testing.

### 2.4. Testing Setup

**Figure 6:**
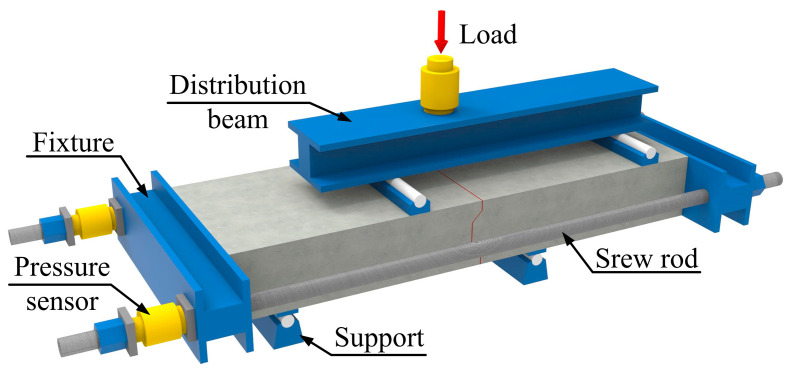
Test loading method.

The test setup used in this study is shown in Figure 6. The specimens were subjected to loading using a hydraulic jack with a maximum capacity of 10,000 kN, which was applied at mid-span above the joint. The load was then transmitted through the beam to both joint sides. As shown in Figure 6, threaded rods were utilized in the precast concrete slabs on both sides of the specimens to apply constraining stresses at the ends. Load sensors were attached to the threaded rods to measure the constraining stress values. Ball-joint steel washers were placed between the sensors and nuts to minimize errors in measuring constraining stress that could be caused by non-axial forces. During the loading process, the load values on the distribution beam were measured by pressure sensors. To investigate the effect of axial constraining forces on the mechanical behavior of the joint, the axial tension force of a threaded rod was applied by tightening a nut. In the test, the axial compressive stresses applied to the precast concrete slabs through the threaded rods were 0 MPa and 2 MPa.

**Figure 7:**
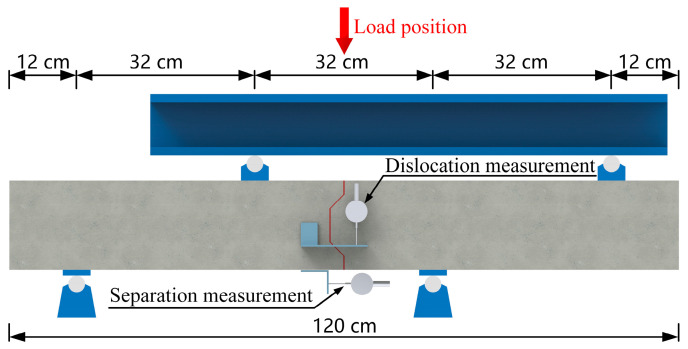
Arrangement of measuring points and fulcrum points.

Detailed information on the load application, supports and measurement point layout are shown in Figure 7. The deformation measurements of the specimens consisted of vertical dislocation and horizontal separation measurements. Two displacement sensors were placed on the front and rear of the specimen to measure displacement values, and the average of the two values was used to represent the vertical dislocation of the precast concrete slabs. Two displacement sensors were also placed at the bottom of the joint to measure the separation values, and the average of the two values was used to represent the horizontal separation of the precast concrete slabs. The loading process of the specimen was strictly performed in stages, with a load interval of 5 kN and a loading rate not exceeding 2 kN/s.

## 3. Experimental Results and Analysis

### 3.1. Failure Mode

**Figure 8:**
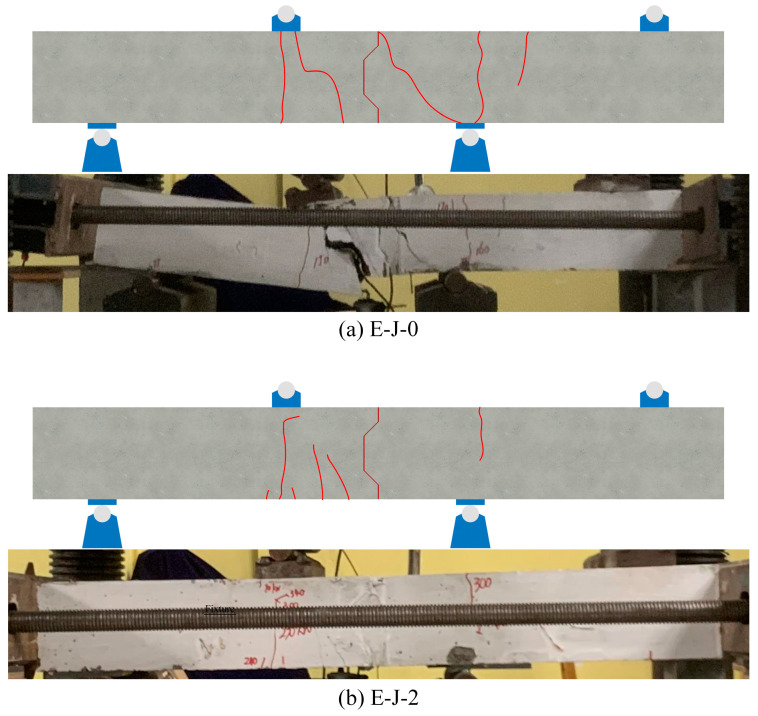
Failure mode of the specimens: (**a**) E-J-0; (**b**) E-J-2.

In the test, shear failure occurred in both specimen E-J-0 and specimen E-J-2, as shown in Figure 8. Cracks appeared on both sides of the mid-span support of the specimens when the load reached 68% and 56% of the peak load, respectively. This indicates that the application of constraint stress caused premature cracking in the specimens. Subsequently, the cracks continued to propagate and increase as the load increased, ultimately leading to the main crack in the specimen distribution of the support area. Although the specimens experienced the process of crack initiation and propagation, there were no significant interface cracks or damage at the joints. After the specimen was damaged, the epoxy joints remained intact, showing strong nodes and weak members.

During the test, both specimens showed slight cracking sounds and caused violent vibrations and loud noises during failure. The main crack developed faster, beginning to extend obliquely from the bottom of the left-side plate until reaching the top of the plate, ultimately leading to shear failure in the specimen. The above results demonstrate the good overall integrity of the specimens, and the epoxy joint can ensure the firm connection of precast concrete panels on both sides. The experimental results are shown in Table 4.

### 3.2. Load–Deflection Relationship

**Figure 9:**
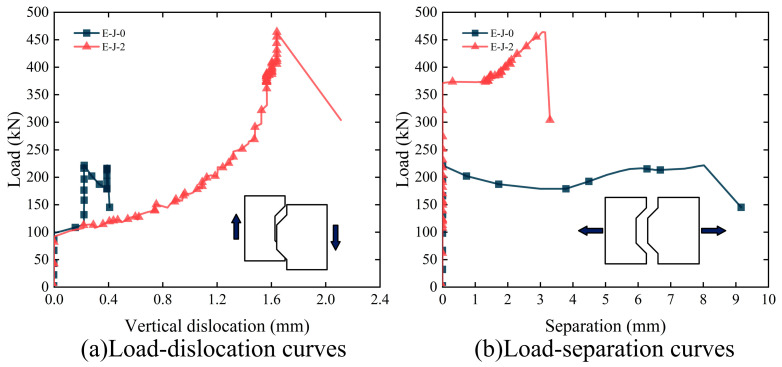
Load–displacement difference curve of epoxy joints: (**a**) load–dislocation curves; (**b**) load–separation curves.

Figure 9 presents the load–displacement curves of the two specimens, which are the vertical displacement shown in Figure 9a and the horizontal separation shown in Figure 9b. The loading process can be divided into three stages. In the first stage, the specimens undergo elastic deformation; the vertical dislocation increases linearly with the increasing applied load; and the vertical dislocation is relatively small. In the second stage, the specimens begin to crack, and the vertical dislocation increases nonlinearly with the increasing applied load until reaching the ultimate load. In the third stage, the load suddenly drops, and the specimens experience shear failure.

In Figure 9, specimen E-J-0 mainly exhibited horizontal separation, while specimen E-J-2 primarily exhibited vertical dislocation. Table 4 shows that, compared with specimen E-J-0, specimen E-J-2 showed increases in the ultimate load of 110.2%, vertical dislocation of 768.4% and a decrease in the horizontal separation of 77.0%. These results indicate that increasing the constraint stress can effectively improve the load-carrying capacity of the specimens and reduce the horizontal separation, but it also leads to increased vertical dislocation at the joint locations.

**Figure 10:**
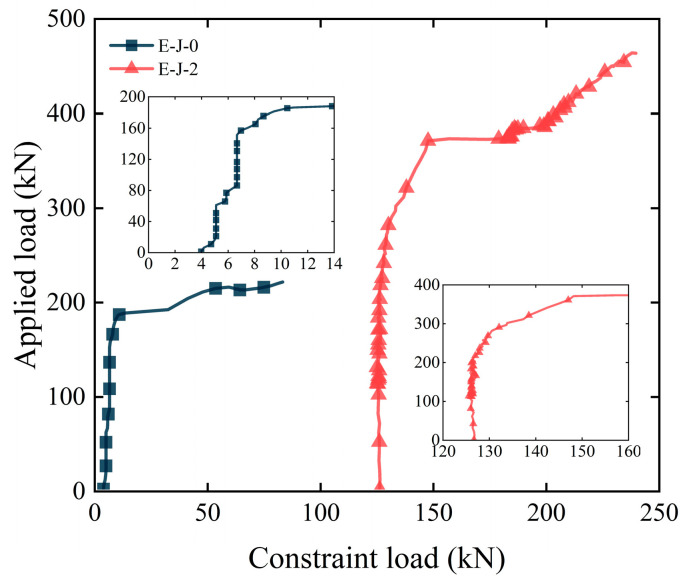
Load-binding force curve of epoxy joints.

Figure 10 presents the load–constraint force curves of two specimens. The initial constraint force of specimen E-J-0 was 4 kN, which generated negligible stress but ensured that the specimen was in a passive constrained state. The initial restraint force of specimen E-J-2 is 128 kN, equivalent to a constraint compressive stress of 2 MPa.

During the early loading stage, the constraint force increased slowly with the increasing load for both specimens. As a significant horizontal separation occurred, the constraint force sharply increased. Combined with the analysis of the load–horizontal separation curve shown in Figure 7b, after the appearance of separation, the constraint force linearly increased with the load because the fixtures on both sides of the specimen constrained its horizontal displacement. In addition, compared with specimen E-J-0, specimen E-J-2 had an ultimate load increase of 110.2% and a maximum constraint force increase of 187.8%. These results indicate that increasing the initial constraint force on both sides of the specimen can limit the lateral deformation of the specimen, thus significantly improving the shear performance of the epoxy joints.

## 4. Finite Element Analysis

### 4.1. Model Establishment

**Figure 11:**
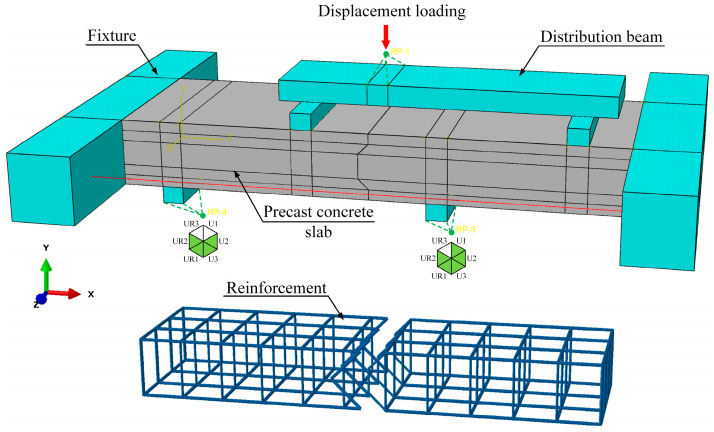
Model composition and its boundary conditions.

To clarify the load-carrying mechanism of epoxy joints between precast concrete slabs, the shear behavior of specimens during loading was simulated using the ABAQUS finite element (FE) software. As shown in Figure 11, the specimen primarily consists of five parts: precast concrete slabs, epoxy joints, a reinforcement cage, distribution beams, and threaded rods. Among them, the precast concrete slabs, epoxy joints, and distribution beams were simulated using C3D8R solid elements with a mesh size of 15 mm. The reinforcement cage and threaded rods were simulated using B31 beam elements with a mesh size of 15 mm and 50 mm, respectively. To save computational cost, the mesh size of the threaded rods was larger. The other parts needed to be observed in more detail, and considering the accuracy of the model and computational efficiency, a mesh size of 15 mm was finally adopted.

The boundary conditions are shown in Figure 11. The left support constrained all degrees of freedom except for U1 and UR3, while the right support only released the UR3 degree of freedom. Furthermore, the constraint force in the threaded rods was realized by equivalent cooling using Equation (1).

The interaction between the different components in the FE model is crucial and challenging. The boundary conditions of the model are shown in Figure 11. The interactions in the model include the interactions between the fixtures, precast concrete slabs and the epoxy joints and the interactions between the reinforcements and the concrete. The epoxy joint between precast concrete slabs simulates the cohesive model. In addition, “embedded” constraints are used to embed the reinforcement cage in the precast concrete slabs. The interaction between the fixtures and precast concrete slabs, as well as between the supports and precast concrete slabs, is modeled using face-to-face contact. The contact characteristics include normal and tangential behavior. The normal behavior is modeled as “hard” contact, where the contact constraints of the corresponding nodes are removed when the interacting surfaces separate. The tangential behavior is defined by a “penalty” function, where the interface friction coefficient is set to 0.6.

(1) $$\Delta T=\frac{F}{E_{s}A_{s}\alpha }$$

In the equation, F denotes the constraint force in the threaded rod. $E_{s}$ and $A_{s}$ are the elastic modulus and cross-sectional area of the threaded rod, respectively. α is the linear expansion coefficient of the steel, which has a value of 1.2 × 10^−5^/°C. Therefore, when the precast concrete slab is passively constrained on both sides, the relative temperature drop of the threaded rod is 1 °C. When the constrained stress on both sides of the precast concrete slab is 2 MPa, the relative temperature drop of the threaded rod is 30 °C.

### 4.2. Material Models

#### 4.2.1. Normal Concrete

**Figure 12:**
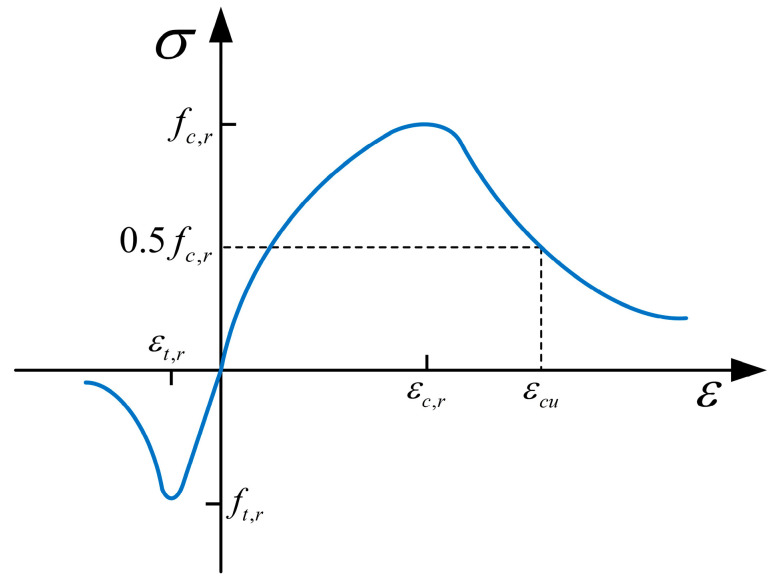
Constitutive relationship model for C50 concrete.

The constitutive model for C50 concrete is based on the stress–strain relationship recommended in the Code for Design of Concrete Structures (GB 50010-2010) [30]. The ultimate compressive strength of C50 concrete is 53.2 MPa. The stress–strain relationship of the concrete shall satisfy Equations (2) and (3), and the curve is shown in Figure 12.

(2) $$\begin{array}{cc} \sigma_{i}=\left(1-d_{i}\right)E_{c}\varepsilon_{i} & i=t,c \end{array}$$

(3) $$\begin{array}{cc} D_{i}=1-\sqrt{\frac{\sigma_{i}}{E_{c}\varepsilon_{i}}} & i=t,c \end{array}$$

In the equation, $\sigma_{i}$ denotes the tensile and compressive stress of concrete, $\varepsilon_{i}$ denotes the tensile and compressive strain of concrete; $E_{c}$ denotes the elastic modulus of concrete; $d_{i}$ denotes the tensile and compressive damage parameters in the constitutive model; and $D_{i}$ denotes the damage factor of concrete in tension and compression required to define the CDP model. Table 5 illustrates the concrete plastic model parameters.

#### 4.2.2. Reinforcement

**Figure 13:**
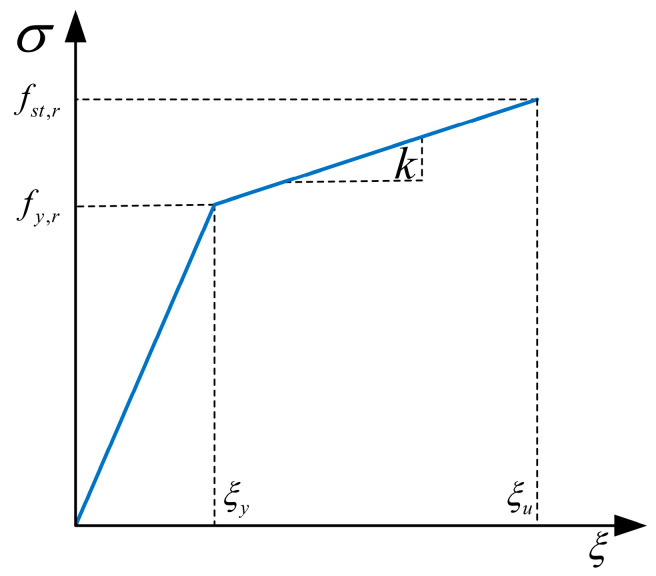
Constitutive relationship model for reinforcement.

All the reinforcements used in the experiment are HRB400, with a yield strength of 401.3 MPa and an ultimate strength of 577.1 MPa. The reinforcement constitutive model adopts the bilinear constitutive model without yield point (Equation (4)) recommended in the Code for the Design of Concrete Structures (GB 50010-2010) [30], as shown in Figure 13.

(4) $$\sigma_{s}=\{\begin{array}{cc} E_{s}\xi_{s} & \xi_{s}\leq \xi_{y}\\ f_{y,r}+k(\xi_{s}-\xi_{y}) & \xi_{y}\leq \xi_{s}\leq \xi_{u}\\ 0 & \xi_{s}\geq \xi_{u} \end{array}$$

In the equation, $E_{s}$ denotes the modulus of elasticity of the reinforcement; $\sigma_{s}$ denotes the stress in the steel bar; $\xi_{s}$ denotes the strain in the steel bar; $f_{y,r}$ denotes the representative value of the yield strength of the steel bar; $\xi_{y}$ denotes the yield strain corresponding to $f_{y,r}$; and k represents the slope of the hardening segment of the reinforcement.

#### 4.2.3. Cohesive Model

**Figure 14:**
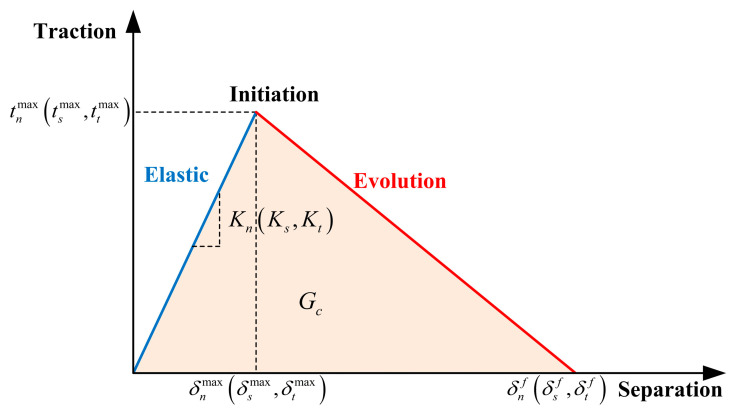
Constitutive relationship model for reinforcement.

The cohesive model is a method used to simulate the bonding interface, which can reveal complex fracture processes between two surfaces through traction–separation relationships. ABAQUS provides two simulation methods for cohesive interfaces: discrete cohesive elements and continuous cohesive elements. In this study, continuous cohesive elements were used to simulate the behavior of the epoxy interface. There are various forms of cohesive models to choose from, and this paper uses the default bilinear relationship model in ABAQUS, as shown in Figure 14 [31].

The contact stresses in the normal, first shear and second shear directions are denoted by $t_{n}$, $t_{s}$ and $t_{t}$, respectively. The stiffnesses in the normal and two tangential directions are represented by $K_{n}$, $K_{s}$ and $K_{t}$. The separations at failure in the normal, first shear and second shear directions are denoted by $\delta_{n}^{\max}$, $\delta_{s}^{\max}$ and $\delta_{t}^{\max}$, respectively. Their values are equal to the ratio of the corresponding strength to stiffness in each direction. Specific parameters are shown in Table 6, where the normal (z) and tangential (x/y) separations correspond to the triaxial coordinates in the FE model. $G_{n}$, $G_{s}$ and $G_{t}$ represent the fracture energy in the normal and tangential directions, respectively.

To simulate the shear behavior of epoxy bond interfaces, zero-thickness cohesive interface elements are employed in the model. The establishment method is as follows: the interface elements are created through offset, based on the surface mesh of the precast plate interface, forming a coincident nodal constraint on one side with the precast plate interface. Moreover, the mesh size of the interface elements is the same size as the precast plate interface mesh.

### 4.3. Model Validation

**Figure 15:**
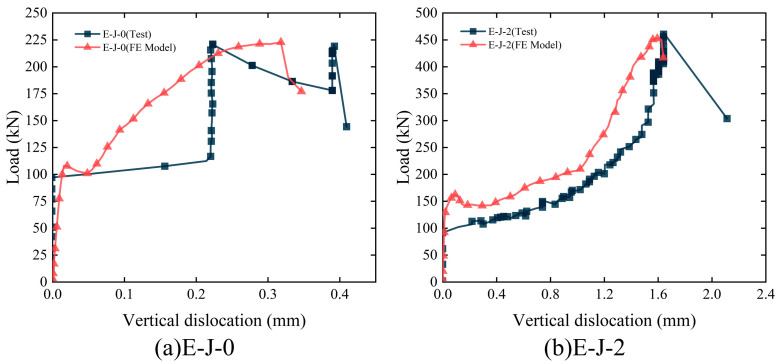
Comparison of load–vertical dislocation curves: (**a**) E-J-0; (**b**) E-J-2.

To verify the effectiveness and accuracy of the FE model, Figure 15 presents a comparison between the FE analysis results and experimental results. Compared with the experimental results, specimens E-J-0 and E-J-2, respectively, exhibit deviations of 3.98% and 2.52% in ultimate load, as calculated using the FE model. For specimen E-J-0, the load–vertical dislocation curve obtained from the FE model is smoother. This could be attributed to the vibrations observed in the experimental specimen during crack initiation, which led to a sudden increase in displacement and resulted in a sawtooth-like curve. However, the FE model fails to accurately simulate this behavior during crack initiation in the specimen. Despite this limitation, the deviations in the load–vertical dislocation curve comparison are relatively small, thereby reflecting the overall trend of the actual loading process of the specimen. For specimen E-J-2, the load–vertical dislocation curves obtained from the experiment and the FE model are nearly identical. In summary, this comparison reveals the limitations of the FE model in simulating specimen behavior, but it also demonstrates that the model can reflect the overall trend of the experimental process, thereby validating its effectiveness and accuracy.

**Figure 16:**
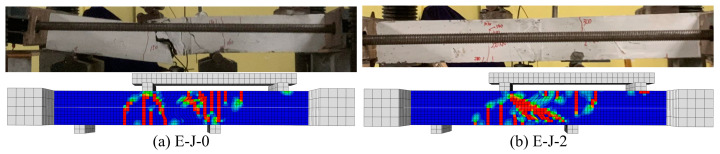
Comparison of test and FE model failure modes: (**a**) E-J-0; (**b**) E-J-2.

In addition, the failure modes of each specimen model were compared with the experimental results. As shown in Figure 16, the crack propagation and failure modes of the specimens are in good agreement with the model. This further confirms the reliability of the model and demonstrates that it is able to predict the behavior of the specimens under load and simulate their failure processes.

### 4.4. Failure Process Analysis

**Figure 17:**
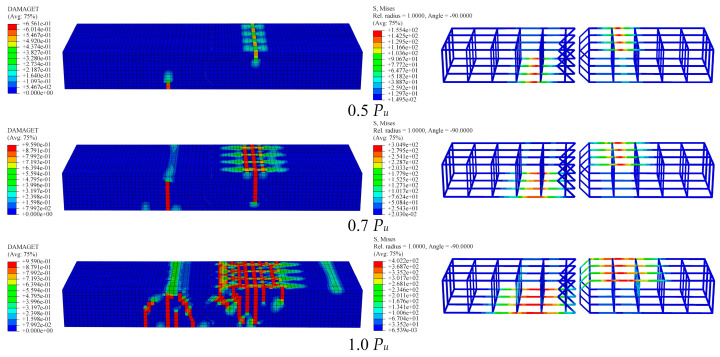
Damage nephograms and reinforcement stress nephograms of precast concrete slabs under different loads.

As shown in Figure 17, this study conducted an analysis of the entire loading process of precast concrete slab epoxy joint specimens under passive confinement conditions, considering concrete tensile damage (DAMAGET) and Mises stress. Under the action of loading, the specimens underwent various stages of deformation and failure. Specifically, during the 0 *P_u_* to 0.5 *P_u_* stage, no tensile cracks appeared in the precast concrete slab, and the reinforcements did not yield. This indicates that the specimen was in the elastic stage. In the 0.5 *P_u_* to 0.7 *P_u_* stage, as the load increased, vertical cracks appeared on the opposite side of the mid-span support, but the transverse reinforcements remained unyielded. This indicates that the specimen entered the stage of working with cracks. With the further increase in load in the 0.7 *P_u_* to 1.0 *P_u_* stage, the number of cracks near the mid-span supports of the specimen started to increase, accompanied by diagonal cracks converging toward the support. Consequently, the longitudinal reinforcement stress on the opposite side of the mid-span support exceeded 401.3 MPa, corresponding to the yield strength. Throughout the entire loading process, the epoxy interface remained intact, indicating the high reliability of the epoxy joint in effectively bearing the load transfer and dispersion of the precast concrete slab in complex loading processes.

**Figure 18:**
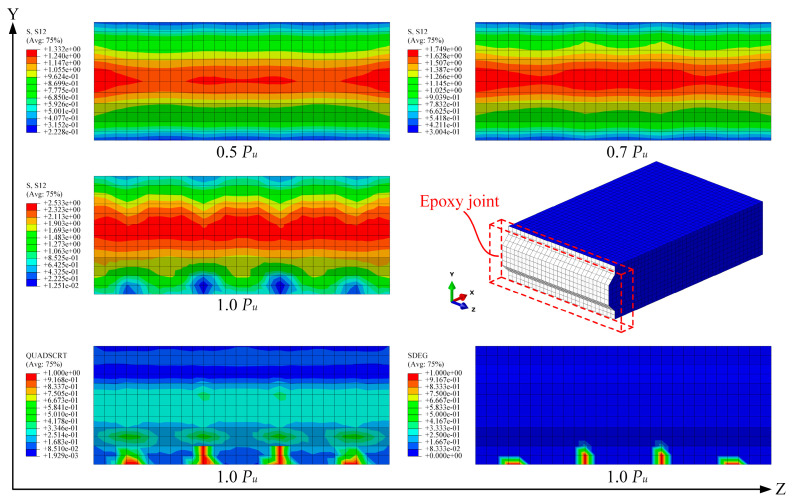
Shear stress distribution and interface damage in epoxy joints under different loads.

The shear stress distribution at the epoxy joint interface under different load levels is illustrated in Figure 18. The shear stresses along the *Y*-axis predominantly concentrate near the shear key region, with a maximum shear stress of 2.53 MPa attained at the ultimate load. The interface damage is quantified by the “SDEG” and “QUADSCRT” indicators, both ranging between 0 and 1, where 0 signifies no damage and 1 represents the complete debonding of the interface. Prior to reaching 1.0 *P_u_*, no damage occurred at the epoxy interface. However, at the 1.0 *P_u_* load level, slight damage was observed at the bottom of the interface, which aligns well with the actual experimental observations. This demonstrates the excellent shear performance of the epoxy joint and indicates that the shear failure of the specimen can be attributed to the precast concrete slab.

### 4.5. Simulation Parameter Analysis

#### 4.5.1. Influence of the Constraint Force

**Figure 19:**
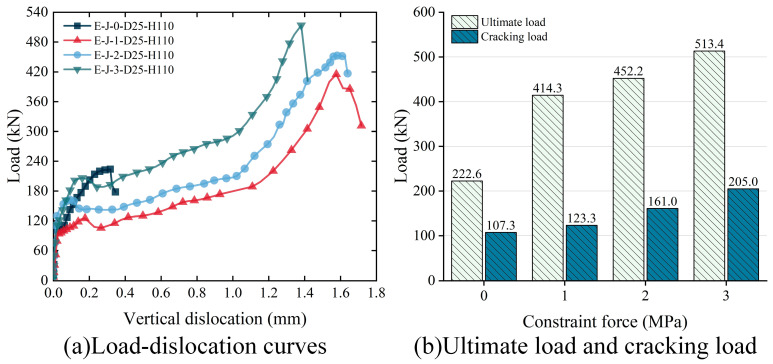
Effect of constraint force on the shear strength of epoxy joints: (**a**) load–dislocation curves; (**b**) ultimate load and cracking load.

Figure 19a compares the load–dislocation curves of the specimens under different levels of constraint. The results reveal that the actively constrained specimens exhibit significantly higher vertical dislocation and ultimate load compared with the passively constrained specimens. Although the constraint force does not significantly alter the shear behavior of the specimens, it still impacts the ultimate load-carrying capacity and vertical displacement. With an increase in the constraint force, the shear resistance gradually increases, while the vertical dislocation slightly decreases.

In order to investigate the influence of constraint force on the shear performance of the specimens, Figure 19b compares the cracking load and ultimate load of the specimens under different levels of constraint. The results indicate that, as the constraint force increases from 0 MPa to 1 MPa, 2 MPa and 3 MPa, the shear capacity of the specimens is enhanced by 86.1%, 103.1% and 130.6%, respectively. The cracking load also increases by 14.9%, 50.0% and 91.1%, respectively. Furthermore, under active constraint conditions, the ratio of the cracking load to the ultimate load increases with the increase in the constraint force.

#### 4.5.2. Influence of the Depth–Height Ratio

**Figure 20:**
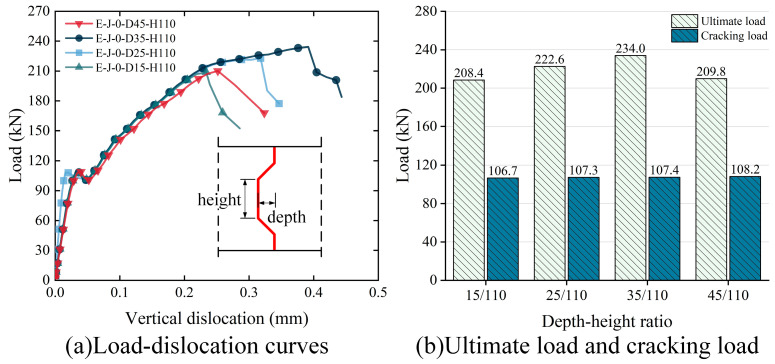
Effect of depth–height ratio on the shear strength of epoxy joints: (**a**) load–dislocation curves; (**b**) ultimate load and cracking load.

Specimen E-J-0-D25-H110 represents the shear key, with a depth of 25 mm and a height of 110 mm, resulting in a depth–height ratio of 25/110. As depicted in Figure 20a, under passive constraint conditions, shear keys with different depth–height ratios exhibit nearly identical shear stiffness. This observation could be attributed to passive constraint weakening the effect of the shear key depth on the shear stiffness of the specimen, indicating the minimal impact of the shear key depth on the shear stiffness. Furthermore, the FE analysis results indicate that increasing the shear key depth does not significantly alter the shear behavior or vertical dislocation of the specimens. The vertical dislocation values remain within the range of 0.2 mm to 0.4 mm.

In order to investigate the influence of the depth–height ratio on the shear performance of specimens, Figure 20b compares the cracking load and ultimate load of specimens with different depth–height ratios. The results reveal that the specimen with a depth–height ratio of 35/110 (E-J-0-D35-H110) exhibits a higher ultimate shear capacity, which is 4.9% to 10.9% higher compared with the other specimens. This finding suggests that a larger depth–height ratio enhances the ultimate shear capacity of the epoxy joint. However, when the depth–height ratio increases to 45/110, the specimen (E-J-0-D45-H110) experiences a significant decrease in ultimate shear capacity. Hence, when the shear key depth–height ratio is 35/110, the specimen exhibits favorable shear resistance. Furthermore, the cracking load of specimens with different depth–height ratios is almost identical, with the differences not exceeding 1.5%.

In summary, the results of the FE analysis indicate that the depth–height ratio of the shear key has a certain influence on the shear performance of epoxy joints. Therefore, when selecting a depth–height ratio, it is necessary to consider the specific conditions of the specimen and conduct a reasonable optimization. Additionally, this study provides a valuable reference for the optimization of the design of epoxy joints based on the specific test results.

#### 4.5.3. Influence of the Interfacial Defect

**Figure 21:**
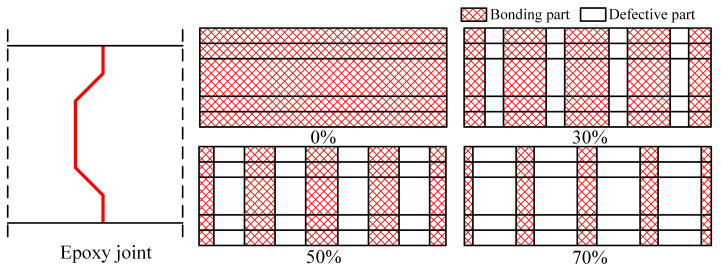
Diagram of interface defects.

In the actual construction process, partial interfaces fail to achieve effective bonding because of factors such as the flow properties of epoxy adhesive, interface impurities or air bubbles and construction errors. Therefore, the relative bonded area becomes a crucial parameter for evaluating the shear performance of epoxy joints. Figure 21 illustrates the specific distribution of interface-bonded areas.

**Figure 22:**
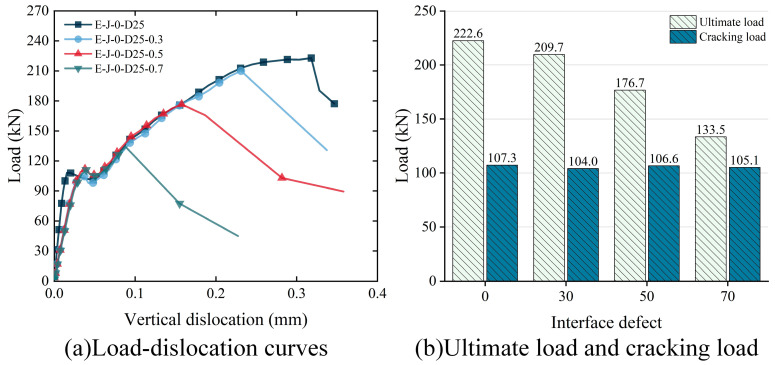
Effect of interfacial defect on the shear strength of epoxy joints: (**a**) load–dislocation curves; (**b**) ultimate load and cracking load.

Through numerical simulation analysis, the influence of different interface defects on the shear performance of epoxy joints has been examined. Figure 22a presents the load–vertical dislocation curves of the specimens under various interface defects. The results indicate that interface defects do not significantly alter the shear behavior of the specimens but have a noticeable effect on their ultimate load-carrying capacity and vertical dislocation. As the interface defect area increases from 0% to 30%, 50% and 70%, the shear load-carrying capacity of the specimens decreases by 5.8%, 20.6% and 40.0%, respectively, while the vertical dislocation decreases by 27.3%, 50.5% and 72.1%, respectively. When the interface defect reaches 30%, there is a shift in the failure mode of the specimens from the shear failure of the prefabricated concrete slab to the interface failure of the epoxy joint. Consequently, larger interface defects result in reduced frictional resistance, leading to the lower shear load-carrying capacity of the specimens, and the ultimate load-carrying capacity is reached before significant dislocation in the prefabricated concrete slab occurs.

To further investigate the influence of interface defects on the shear performance of the specimens, Figure 22b compares the cracking load and the ultimate load of the specimens with different interface defect areas. The results indicate that the shear load-carrying capacity of the specimens decreases linearly with an increase in the interface defect area. However, there is no significant impact on the cracking load of the specimens, with a difference range of less than 3.5%. This finding provides reference data for further research and improvement of epoxy joint construction.

## 5. Conclusions

The main conclusions are as follows:

1. The utilization of the single-shear test method in this study has effectively demonstrated the capacity to reflect the shear performance of transverse joints in precast bridge deck panels under short-term loads. Both scenarios involving passive and active restraints yielded excellent shear performance results for the epoxy joints. No emergence of interface cracks within the epoxy joints was detected throughout the loading processes applied to the specimens.
2. The shear failure process of the specimens can be divided into three stages: an elastic stage, a cracking stage and a failure stage. A considerable degree of overall integrity was maintained by the specimens during the failure process, made possible by the epoxy joints.
3. When the constraint force was increased from 0 MPa to 2 MPa, the ultimate load of the test specimens increased by 110.2%; the vertical displacement increased by 768.4%; and the horizontal separation decreased by 77.0%. Therefore, increasing the constraint force can effectively enhance the shear-bearing capacity of the specimens and reduce the occurrence of horizontal separation, but it will result in increased vertical dislocation at the joint.
4. The application of constraint force significantly enhances the shear performance of epoxy joints. Compared with the passive restraint specimens, the active restraint specimens exhibit an improved shear-carrying capacity ranging from 86.1% to 130.6%.
5. The shear strength of epoxy joints with a depth–height ratio of 35/110 increased by 4.9% to 10.9% compared with those with ratios of 15/110, 25/110 and 45/110. Moreover, as the interface defects increased from 0% to 70%, the shear-carrying capacity of epoxy joints was reduced by 5.8% to 40%. When the interface defects in the epoxy joints exceeded 30%, the failure mode shifted from shear failure to interface failure.

## Figures and Tables

**Table 1:** Design details of specimens.

Specimen	Joint Type	Restraint Stress (MPa)	Interface Treatment
E-J-0	Epoxy	0	Smooth
E-J-2	Epoxy	2	Smooth

**Table 2:** Mechanical properties of epoxy.

Material	$f_{te}\;(\mathrm{MPa})$	$f_{ce}\;(\mathrm{MPa})$	$E_{te}\;(\mathrm{MPa})$
CFSR-A/B	38	70	240

**Table 3:** Mechanical properties of reinforcement and normal concrete materials.

Material	$f_{c}$ (MPa)	$E_{c}\;(\mathrm{GPa})$	$f_{y}\;(\mathrm{MPa})$	$f_{s}\;(\mathrm{MPa})$	$E_{s}\;(\mathrm{GPa})$
C50	53.2	35.1	-	-	-
HRB400	-	-	401.3	577.1	203.7

**Table 4:** Test results.

Specimen	*P_u_* (kN)	*Y_u_* (kN)	*S_u_* (mm)	*Z_u_* (mm)	Failure Mode
E-J-0	220.6	129.8	0.19	13.9	Shear failure
E-J-2	463.7	240.6	1.65	3.2	Shear failure

Note: *P_u_* is the ultimate load of each specimen; *Y_u_* is the maximum constraint force at both ends of the precast concrete panel; *S_u_* is the maximum vertical dislocation value on both sides of the joint; *Z_u_* is the maximum horizontal separation value at the bottom of the joint.

**Table 5:** Plasticity parameters for concrete materials.

Dilation Angle ψ	Eccentricity λ	Yield Stress Ratio $\sigma_{bo}/\sigma_{co}$	$\mathrm{Constant}\;\mathrm{Stress}\;\mathrm{Ratio}\;K_{c}$	Viscosity Parameter
30°	0.1	1.16	0.6667	0.00005

**Table 6:** Cohesive model parameters of epoxy interface.

Direction	$t_{n,s,t}\;(\mathrm{MPa})$	$K_{n,s,t}\;(\mathrm{MPa})$	$G_{n,s,t}\;(\mathrm{MPa})$
Normal (z)	2.388	199	0.0129
Tangent (x)	4.356	453.75	0.027444
Tangent (y)	4.356	453.75	0.027444

## Data Availability

The original data of the results of this study are archived. If necessary, the editorial department can contact jinlongjiang@mails.cqjtu.edu.cn by email.

## Abbreviations

List of Symbols	
$f_{te}$	Tensile strength of epoxy
$f_{ce}$	Compressive strength of epoxy
$E_{te}$	Tensile elastic modulus of epoxy
$f_{c}$	Compressive strength of concrete
$E_{c}$	Tensile elastic modulus of concrete
$f_{y}$	Yield strength of steel
$f_{s}$	Ultimate strength of steel
$E_{s}$	Tensile elastic modulus of steel
$P_{u}$	Ultimate load of each specimen
$Y_{u}$	Maximum constraint force at both ends of the precast concrete panel
$S_{u}$	Maximum vertical dislocation value on both sides of the joint
$Z_{u}$	Maximum horizontal separation value at the bottom of the joint
$A_{s}$	Cross-sectional area
α	Linear expansion coefficient of the steel
$\sigma_{i}$	Tensile and compressive stress of concrete
$\varepsilon_{i}$	Tensile and compressive strain of concrete
$d_{i}$	Tensile and compressive damage parameters in the constitutive model
$D_{i}$	Damage factor of concrete in tension and compression
ψ	Dilation angle
λ	Eccentricity
$\sigma_{bo}/\sigma_{co}$	Yield stress ratio
$K_{c}$	Constant stress ratio
$\xi_{s}$	Stress of steel bar
$f_{y,r}$	Representative value of the yield strength of the steel bar
$\xi_{y}$	Yield strain of steel bar
k	Slope of the hardening segment of the reinforcement
$t_{n}$	Contact stresses in the normal
$t_{s}$	Contact stresses in the first shear direction
$t_{t}$	Contact stresses in the second shear direction
$K_{n}$	Stiffness in the normal
$K_{s}$	Stiffness in the first shear direction
$K_{t}$	Stiffness in the second shear direction
$\delta_{{}_{n}}^{\max}$	Separations at failure in the normal
$\delta_{{}_{s}}^{\max}$	Separations at failure in the first shear direction
$\delta_{{}_{t}}^{\max}$	Separations at failure in the second shear direction
$G_{n}$	Fracture energy in the normal
$G_{s}$	Fracture energy in the first shear direction
$G_{t}$	Fracture energy in the second shear direction

## References

[1] Yang T., Liu S.Y., Qin B.X., Liu Y.Q. (2020). Experimental study on multi-bolt shear connectors of prefabricated steel-concrete composite beams. J. Constr. Steel Res..

[2] Zou Y., Qin F.J., Zhou J.T., Zheng Z.C., Huang Z.L., Zhang Z.Y. (2021). Shear behavior of a novel bearing-shear connector for prefabricated concrete decks. Constr. Build. Mater..

[3] Luo Y.-B., Yan J.-B. (2022). Developments of prefabricated steel-concrete composite beams with novel steel-yielding demountable bolt connectors. J. Constr. Steel Res..

[4] Kagioglou P., Katakalos K., Mitoulis S.A. (2021). Resilient connection for accelerated bridge constructions. Structures.

[5] Terreros-Bedoya A., Negrin I., Payá-Zaforteza I., Yepes V. (2023). Hybrid steel girders: Review, advantages and new horizons in research and applications. J. Constr. Steel Res..

[6] Jia J., Ren Z., Bai Y., Li J., Li B., Sun Y., Zhang Z., Zhang J. (2023). Tensile behavior of UHPC wet joints for precast bridge deck panels. Eng. Struct..

[7] Han B., Zuo Z., Di J., Qin F. (2021). Flexural behaviour of improved joint details connecting pre-cast bridge decks. Eng. Struct..

[8] Hu M., Jia Z., Han Q., Ni Y., Jiao C., Long P. (2022). Shear behavior of innovative high performance joints for precast concrete deck panels. Eng. Struct..

[9] Shao Z., Huai C., Cao J., Li C., Shao X. (2022). Experimental investigation and design optimization on flexural behavior of new UHPC deck panel with longitudinal ribs reinforced by steel plates. Structures.

[10] Di J., Han B., Qin F. (2020). Investigation of U-bar joints between precast bridge decks loaded in combined bending and shear. Structures.

[11] Deng E.-F., Zhang Z., Zhang C.-X., Tang Y., Wang W., Du Z.-J., Gao J.-P. (2023). Experimental study on flexural behavior of UHPC wet joint in prefabricated multi-girder bridge. Eng. Struct..

[12] Ahmed G.H., Aziz O.Q. (2019). Shear strength of joints in precast posttensioned segmental bridges during 1959–2019, review and analysis. Structures.

[13] Arafa A., Farghaly A.S., Ahmed E.A., Benmokrane B. (2016). Laboratory testing of GFRP-RC panels with UHPFRC joints of the nipigon river cable-stayed bridge in northwest ontario, Canada. J. Bridge Eng..

[14] Verger-Leboeuf S., Charron J.-P., Massicotte B. (2017). Design and behavior of UHPFRC field-cast transverse connections between precast bridge deck elements. J. Bridge Eng..

[15] Ahmed G.H., Aziz O.Q. (2019). Influence of intensity & eccentricity of posttensioning force and concrete strength on shear behavior of epoxied joints in segmental box girder bridges. Constr. Build. Mater..

[16] Zou Y., Jiang J., Yang J., Zhang Z., Guo J. (2023). Enhancing the toughness of bonding interface in steel-UHPC composite structure through fiber bridging. Cem. Concr. Compos..

[17] Gopal B.A., Hejazi F., Hafezolghorani M., Voo Y.L. (2020). Shear Strength of Dry and Epoxy Joints for Ultra-High-Performance Fiber-Reinforced Concrete. Struct. J..

[18] Zhang Y., Zhang Z., Hu F., Du X., Lu Y., Zhu J. (2022). Full-scale experimental study on shear behavior of multiple-keyed epoxy joints in precast concrete segmental bridges. Structures.

[19] Yuan A., Yang C., Wang J., Chen L., Lu R. (2019). Shear Behavior of Epoxy Resin Joints in Precast Concrete Segmental Bridges. J. Bridge Eng..

[20] Chen L., Yan J., Xiang N., Zhong J. (2022). Shear performance of ultra-high performance concrete multi-keyed epoxy joints in precast segmental bridges. Structures.

[21] Ye M., Li L., Li H., Zhou C. (2022). Shear behavior of joints in precast UHPC segmental bridges under direct shear loading. Constr. Build. Mater..

[22] Pan R., Cheng L., He W., Zhou X., Shen X. (2022). Direct shear performance of UHPC Multi-Keyed epoxy joint. Structures.

[23] Zou Y., Xu D. (2022). Experimental study on shear behavior of joints in precast concrete segmental bridges. Structures.

[24] Jiang H., Huang C., Mei G., Gao X., Tian Y., Sun X. (2023). Experimental and numerical investigations on direct shear performance of UHPC dry joints. Eng. Struct..

[25] Qiu M., Shao X., Yan B., Zhu Y., Chen Y. (2022). Flexural behavior of UHPC joints for precast UHPC deck slabs. Eng. Struct..

[26] Feng Z., Li C., Ke L., Yoo D.-Y. (2022). Experimental and numerical investigations on flexural performance of ultra-high-performance concrete (UHPC) beams with wet joints. Structures.

[27] (2021). Test Methods for Properties of Resin Casting Body.

[28] (2002). Standard for Test Method of Performance on Ordinary Fresh Concrete.

[29] (2010). Metallic Materials-Tensile Testing-Part 1: Method of Test at Room Temperature.

[30] (2010). Code for Design of Concrete Structures.

[31] Ranz D., Cuartero J., Castejon L., Miralbes R., Malon H. (2020). A cohesive zone model approach to interlaminar behaviour of carbon/epoxy laminated curved beams. Compos. Struct..

